# Optimizing methods for PCR-based analysis of predation

**DOI:** 10.1111/j.1755-0998.2011.03018.x

**Published:** 2011-09

**Authors:** Daniela Sint, Lorna Raso, Rüdiger Kaufmann, Michael Traugott

**Affiliations:** Institute of Ecology, University of InnsbruckTechnikerstraße 25, 6020 Innsbruck, Austria

**Keywords:** annealing temperature, molecular gut content analysis, *Oreonebria*, *Pardosa*, replicability, visualization methods

## Abstract

Molecular methods have become an important tool for studying feeding interactions under natural conditions. Despite their growing importance, many methodological aspects have not yet been evaluated but need to be considered to fully exploit the potential of this approach. Using feeding experiments with high alpine carabid beetles and lycosid spiders, we investigated how PCR annealing temperature affects prey DNA detection success and how post-PCR visualization methods differ in their sensitivity. Moreover, the replicability of prey DNA detection among individual PCR assays was tested using beetles and spiders that had digested their prey for extended times postfeeding. By screening all predators for three differently sized prey DNA fragments (range 116–612 bp), we found that only in the longest PCR product, a marked decrease in prey detection success occurred. Lowering maximum annealing temperatures by 4 °C resulted in significantly increased prey DNA detection rates in both predator taxa. Among the three post-PCR visualization methods, an eightfold difference in sensitivity was observed. Repeated screening of predators increased the total number of samples scoring positive, although the proportion of samples testing positive did not vary significantly between different PCRs. The present findings demonstrate that assay sensitivity, in combination with other methodological factors, plays a crucial role to obtain robust trophic interaction data. Future work employing molecular prey detection should thus consider and minimize the methodologically induced variation that would also allow for better cross-study comparisons.

## Introduction

Molecular techniques are currently being widely employed to study food web interactions in both aquatic and terrestrial systems as they allow feeding to be tracked at high specificity and sensitivity (Symondson 2002; Sheppard & Harwood 2005; Gariepy *et al.* 2007). They open a variety of new opportunities in trophic ecology, but methodological issues still represent an important point for the further development of this approach (King *et al.* 2008). It has been shown that environmental factors such as ambient temperature (McMillan *et al.* 2007; von Berg *et al.* 2008; Hosseini *et al.* 2008) or the type and amount of ingested prey as well as the species of predator (e.g. Sheppard *et al.* 2005; Greenstone *et al.* 2007; Traugott & Symondson 2008) can influence postfeeding prey detection periods in arthropod predators. Aside from these field parameters, a range of methodological aspects including, for example, field sampling techniques (Harwood 2008; Chapman *et al.* 2010; Greenstone *et al.* 2011), sample washing (Remén *et al.* 2010) and preservation (Weber & Lundgren 2009), DNA extraction protocols (Oehm *et al.* 2011) and size-dependent differences in prey amplicon detection success (e.g. Hoogendoorn & Heimpel 2001; Traugott & Symondson 2008) need to be carefully considered for work that utilizes PCR-based analysis of predation. Other factors such as the sensitivity of DNA visualization methods or the replicability of diagnostic PCR results have not yet been evaluated. The sensitivity of a prey DNA detection systems and the replicability of the screening results, however, can be important sources for variation and have a considerable effect on the outcome of a study including the alteration of the conclusions drawn from the results obtained. As studies usually vary in the methodological issues highlighted earlier, knowing the methodological variability is essential when comparing different studies, as only then it is possible to rate differences and estimate whether they are within an expected range of variation.

Here, we investigate methodological parameters influencing prey DNA detection limits to optimize PCR-based gut content analysis and hence to minimize variation introduced by methodology. Based on feeding experiments with two cold-adapted predator taxa commonly found in high alpine areas—carabid beetles and lycosid spiders—we test how PCR annealing temperature and post-PCR visualization methods affect prey DNA detection success for three differently sized PCR products. Furthermore, we examine the replicability of prey DNA detection success for beetles and spiders at extended times postfeeding, a situation when predators are likely to contain only minute amounts of prey DNA, and therefore, variation in prey detection is expected to be high.

## Material and methods

### Origin of predators

In July 2008, 70 lycosid spiders (adults and juveniles of both sexes of *Pardosa nigra* (C.L. Koch, 1834) and *P. saturatior* Simon, 1937) and 71 carabid beetles [10 *Nebria germari* Heer, 1837 and 61 *Oreonebria castanea* (Bonelli, 1810)] were collected in Gaisbergtal (Ötztal, Tyrol, Austria) at 2500 m a.s.l. in the glacier foreland of the ‘Gaisbergferner’ (WGS84: N 46.837°, E 011.054°). The two species of carabid beetles were pooled for the feeding experiments, as they are closely related, of equal size and live under the same environmental conditions, so they can be expected to have similar digestion rates, similar to closely related spiders where prey protein digestion rates were found not to be different (Harwood *et al.* 2004, 2005).

### Feeding experiments

Prior to the feeding experiments, all animals were kept individually in a climate chamber (14:10 L:D) and starved for a minimum of 1 week to allow digestion of any food they had consumed before being captured as well as to adjust them to a similar hunger level. The temperature was set to 10 °C, the daily mean temperature at 2500 m a.s.l. as measured in the neighbouring valley of Gaisbergtal during the collection period.

After the starvation period, carabid beetles were provided with 1/3 of a freeze-killed mealworm (*Tenebrio molitor*), allowed to feed for 2 h in the dark as they are night-active and then kept at 10 °C at 14:10 L:D. At 0, 8, 16, 24, 32, 40 and 48 h postfeeding, 10 randomly chosen individuals (11 after 48 h; min 1 *N. germari* at each time point) were freeze-killed.

As the alpine spiders used in this experiment are day-active and hunt in bright sunlight on warm surfaces (pers. observations of authors), they were fed in the light at 15 °C. One small, live cricket (*Acheta domesticus*) was offered to each spider, which was then allowed to feed for a maximum of 2 h (less if a spider had finished its meal before). After feeding, spiders were kept again at 10 °C and 14:10 L:D. Only 60 individuals could be used for the experiments as some spiders did not feed, leaving between eight and 10 individuals each to be frozen at 0, 16, 32, 48, 60, 72 and 84 h postfeeding. Extended digestion times were chosen for spiders as in previous studies, prolonged prey DNA detection success was found in liquid feeders (Ma *et al.* 2005; Greenstone *et al.* 2007; Traugott & Symondson 2008).

After commencement of the experiment, all predators were stored at −28 °C until extracting their DNA from the whole animal using a modified CTAB protocol (Juen & Traugott 2005) and subsequent DNA purification using the Geneclean Turbo Kit (Qbiogene, Inc., Carlsbad, California) following the manufacturer’s instructions. Negative controls (i.e. no animal tissue) were included in each batch of samples to check for potential DNA carry-over contamination during extraction.

### Molecular analysis of feeding experiments

A ∼700-bp fragment of the mitochondrial cytochrome c oxidase subunit one gene (COI) was sequenced for crickets (GenBank accession no. JF419327) to develop prey-specific primer pairs following standard procedures described earlier (King *et al.* 2008). For the detection of mealworm DNA, a primer pair amplifying a 128-bp fragment was already available (Oehm *et al.* 2011); thus, only two additional sense primers were designed based on GenBank accession nos.HQ891143–HQ891145. The final primer sets allowed amplifying fragments of three different lengths for both species (Table 1).

**Table 1:** Primer pairs designed from COI mtDNA sequences of mealworm (*Tenebrio molitor*) and cricket (*Achaeta domesticus*). Columns show the primer targets, primer names (S and A denotes forward and reverse primers, respectively), primer sequences and the expected product size. Primers S210 and A212 (Oehm *et al.* 2011) and all other primers present study are also shown

Target species	Primer names and sequences (5′–3′)	Size (bp)
*T. molitor*	S210: TACCGTTATTCGTATGAGCAGTAT	128
	A212: CGCTGGGTCAAAGAAGGAT	
	S232: TAATAAGAAGAATTGTAGAAAACGGG	332
	A212: CGCTGGGTCAAAGAAGGAT	
	S231: TCATTTTTGGAGCGTGATCC	612
	A212: CGCTGGGTCAAAGAAGGAT	
*A. domesticus*	S228: CACCCTCACTAACCCTTTTATTAACC	116
	A231: ATCAACAGATGCTCCGGCG	
	S228: CACCCTCACTAACCCTTTTATTAACC	350
	A233: GGGGTCAAAAAATGATGTATTCAG	
	S230: CGAACGGAACTAGGACAACCA	555
	A233: GGGGTCAAAAAATGATGTATTCAG	

After gradient PCR and verification that no predator DNA could be amplified, the primers were used to screen the animals from the feeding experiments for their prey. Each 10-μL PCR mix contained 1.5 μL of extracted and purified DNA, 1× PCR Buffer (Genecraft, Cologne, Germany), 1 μm of each primer, 0.2 mm dNTPs (Genecraft), 3 mm MgCl_2_, 10 μg bovine serum albumin and 0.375 U *Taq* polymerase (Genecraft). Thermal cycling included 2 min at 94 °C, 35 cycles of 20 s at 94 °C, 30 s at 64–69 °C, 1 min at 72 °C and final elongation for 3 min at 72 °C. It has been suggested to use the highest possible annealing temperature to maximize primer specificity in prey detection assays (King *et al.* 2008). To evaluate the effect of a high annealing temperature on the sensitivity of the PCR assays, the primer pairs were tested at different annealing temperatures. While the primer combinations for the medium and long fragment allowed testing at 65 and 69 °C, the shortest fragments were tested at their highest possible annealing temperature of 66 °C (mealworm) and 64 °C (cricket). Negative controls (no DNA template) were included in each PCR to detect potential carry-over contamination of DNA.

PCR products were separated and visualized using QIAxcel (an automated capillary electrophoresis system by Qiagen, Hilden, Germany) with the QIAxcel DNA Screening Kit and method AL320 (sample uptake 20 s at 8 KV, separation 320 s at 6 KV). Electropherograms were analysed with BioCalculator 3.0 (Qiagen), and all samples that generated a fragment of the expected length and yielded ≥0.1 relative fluorescent units (RFU) were counted as positive.

### Sensitivity of post-PCR visualization methods

QIAxcel and standard agarose gel electrophoresis using either gels stained with ethidium bromide or GelRed™ (Biotium, Hayward, USA) were compared for their sensitivity to visualize PCR products. PCRs with the same conditions as in the feeding experiment were conducted for all three fragment sizes, using pure *A. domesticus* DNA as template. After amplification, the different fragments were mixed and then diluted stepwise 1:2 with QX DNA Dilution Buffer (Qiagen). Dilutions were run on QIAxcel [QIAxcel DNA High Resolution Kit; method OL400 (sample uptake 20 s at 8 KV, separation 400 s at 6 KV)]. Three μL each of the same sample was loaded onto 1.5% agarose gels stained with either ethidium bromide (conc. 0.33 μg/mL) or 1x GelRed™ and separated for 60 min at 7 V/cm.

### Replicability of prey detection

To test the variability in prey DNA detection success in individual samples, DNA extracts of both spiders (*n* = 18) and beetles (*n* = 21), which digested their meals up to the latest two time points postfeeding, were re-tested four times. The test was carried out in different PCRs on different days using the primer pairs resulting in the longest prey fragment (555 and 612 bp for spiders and beetles, respectively) at an annealing temperature of 65 °C following the PCR protocol and the QIAxcel procedures described earlier. Only predators that had digested their prey for extended times postfeeding were considered here as these are likely to contain only minor quantities of the long prey DNA fragments (Deagle *et al.* 2006), a situation where the variability in prey DNA detection success might strongly influence prey DNA detection rates.

All reactions generating ≥0.1 RFU in the electropherogram were counted as positive and given a value of 1. In case a clear peak was visible by eye, but did not reach 0.1 RFU, the sample was counted as weak and got a value between 0.01 and 0.99 relative to the peak height (percentage of peak height from 0.1 RFU). If no clear peak was visible (usually ‘peaks’ below 0.03 RFU could not be separated from the cartridge’s background noise), the reaction was counted as negative and got a value of 0.

### Statistical analyses

To compare prey DNA detection success over time, LOGIT analysis was carried out using spss for both predator taxa. The time point for a prey detection probability of 50 % was determined and comparisons between carabids and lycosids were based on the 95 % confidence limits (cl). Nonparametric McNemar test was used to test for significant differences in prey detection success between higher and lower annealing temperature as well as for evaluating the increase in samples testing positive after five PCRs.

To test for significant differences in prey detection success between the repeated PCRs, 95% tilting confidence limits were calculated from 9999 bootstrap resamples using S-Plus 8.0 (TIBCO Software Inc., Paolo Alto, USA). Nonoverlapping confidence limits were interpreted as significant differences.

## Results

### Detecting prey DNA at different time points postfeeding

**Fig. 1:**
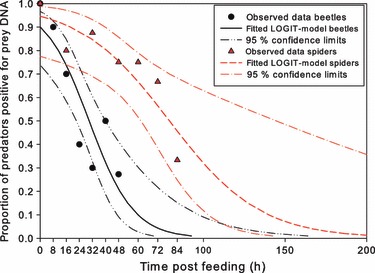
Prey DNA detection success in alpine carabid beetles fed with mealworms and lycosid spiders fed with crickets at different time points postfeeding for long prey DNA fragments (mealworms 612 bp, crickets 555 bp) and fitted LOGIT models with 95 % confidence limits.

Prey DNA detection success in both beetles and spiders was dependent on the length of the fragment targeted in PCR. The shortest fragments could be detected in 100 % of the animals at all time points, and the medium-sized fragments were missed only in three of the spiders (one at 16 h and two at 84 h). The detection success of the longest prey amplicon showed a decline over time from 100 % to 27.3 % at 48-h postfeeding in beetles and 33.3 % at 84-h postfeeding in spiders (Fig. 1). Both LOGIT models described this decline adequately (Pearson Chi-square for beetles and spiders were χ²(5) = 6.050 at *P* = 0.301 and χ²(5) = 3.074 at *P* = 0.689, respectively). Based on these models, the probability of a 50 % prey detection success was reached after significantly different time spans for the two predator taxa: in beetles, 30.0 h (cl: 22.2 and 40.0 h) and in spiders, 79.2 h (cl: 61.8 and 144.5 h) (Fig. 1).

**Fig. 2:**
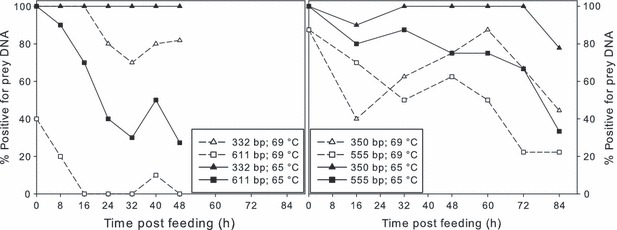
Prey DNA detection success in carabid beetles (left) and lycosid spiders (right) for medium-sized and long prey DNA fragments at different annealing temperatures (65 and 69 °C).

Increasing the annealing temperature from 65 to 69 °C (which was the highest possible temperature determined by gradient PCR with pure prey DNA) resulted in a considerable drop in prey DNA detection success for the medium- and long-sized fragments in both beetles and spiders (Fig. 2). Considering all samples within a feeding experiment, the McNemar test showed that the numbers of samples testing positive for prey DNA were significantly higher at 65 °C in spiders (long and medium fragment: *P* <0.001) and beetles (long fragment: *P* <0.001, medium fragment: *P* <0.01).

### Sensitivity of post-PCR visualization methods

**Fig. 3:**
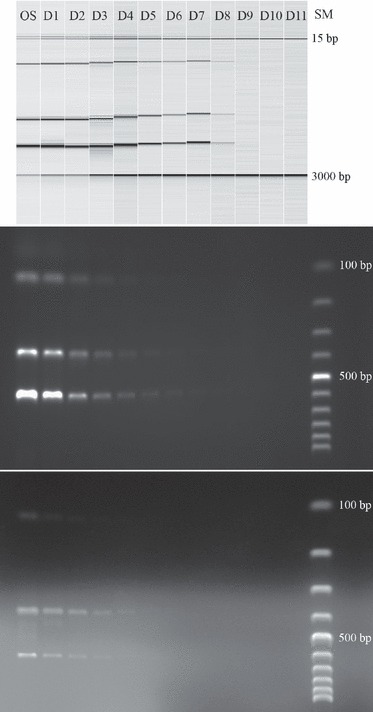
Serial dilution (1:2) of a mixture of three *Acheta domesticus* DNA fragments (116, 350, 555 bp) visualized with the QIAxcel system (top), GelRed™—(middle) and ethidium bromide-stained agarose gels (bottom). OS: original sample, D1—D11: dilution steps, SM: size marker (100–1000 bp; note: with QIAxcel, an internal marker (15 and 3000 bp) is used).

When comparing the three visualization techniques, both QIAxcel and agarose gels stained with GelRed™ were superior to ethidium bromide-stained agarose gels. Fig. 3 shows a serial dilution of a mixture of three DNA fragments separated and visualized by the three methods. QIAxcel and GelRed™ show nearly the same resolution. With QIAxcel, all fragments can be detected to the 8th dilution step, which corresponds to a DNA concentration that is 1/256th or 0.0039 of the original concentration of the fragments. With GelRed™, one fragment still was detectable at the 7th dilution step (1/128th), whereas on a gel stained with ethidium bromide, products could be visualized up to the 4th dilution step only, corresponding to 1/16th of the original DNA concentration (Fig. 3).

### Replicability of prey detection

**Fig. 4:**
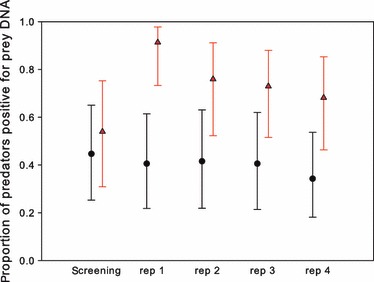
Prey DNA detection success for 21 beetles (circles) and 18 spiders (triangles) in five individual PCRs (original screening + 4 repetitions) with their 95 % tilting confidence intervals obtained from 9999 bootstrap resamples. Animals were allowed to digest their meal for extended time spans i.e. 40 or 48 h (beetles) and 72 or 84 h (spiders).

From the 39 predators (21 beetles and 18 spiders) that had digested their meals for extended times postfeeding and which were screened five times for the presence of the longest prey DNA fragments, 51.2 % were constantly positive or negative. Another 20.6 % tested weak (<0.1 RFU)/positive (≥0.1 RFU) or weak/negative (no peak) and 28.2 % gave results varying from positive to negative (Table 2). The proportion of samples testing positive did not vary significantly between the different PCRs in beetles and spiders (Fig. 4).

**Table 2:** Numbers (%) of predators that show different combinations of prey DNA detection levels when tested five times under the same PCR conditions. Detection levels are positive [peak in the electropherogram ≥0.1 relative fluorescent units (RFU)], weak (peak <0.1 RFU) and negative (no clear peak visible)

	Always positive (%)	Positive + weak (%)	Positive + weak + negative (%)	Positive + negative (%)	Weak + negative (%)	Always negative	Sum (%)
Beetles	3 (14.3)	3 (14.3)	3 (14.3)	1 (4.8)	1 (4.8)	10 (47.6%)	21 (100)
Spiders	7 (38.9)	1 (5.6)	3 (16.7)	4 (22.2)	3 (16.7)	0	18 (100)
Total	10 (25.6)	4 (10.3)	6 (15.4)	5 (12.8)	4 (10.3)	10 (25.6%)	39 (100)

The cumulative total number of positive samples (i.e. ≥0.1 RFU in at least 1 PCR) increased from 8 (38.1 %) after the first PCR to 10 (47.6 %) after five PCRs in beetles and from 9 (50.0 %) to 16 (88.9 %) in spiders. This increase was significant for spiders (McNemar, *P* < 0.01) but not for beetles. In both predator groups, the number of samples testing positive did not increase further after three consecutive PCRs.

## Discussion

Within the current experiments, we found that the annealing temperature used in a PCR assay can greatly affect detection success of prey DNA. An increase of 4 °C (from 65 to 69 °C) resulted in a 47.9%/12.7% and 21.7%/28.3% drop in overall prey DNA detection for beetles and spiders targeting the long/medium fragment, respectively. We ascribe this phenomenon to a significantly reduced number of primers that can anneal to the target DNA at high compared with the lowered annealing temperature. Thus, to increase the sensitivity of PCR assays in prey detection studies, we suggest not to use the highest annealing temperature that allows an amplicon to be obtained, but instead to decrease it to a level where the specificity for the assay still is assured, which is a modification of the recommendation given in King *et al.* (2008).

The present data show that visualization methods for PCR products pose another source of variability for molecular diagnostics such as trophic interaction studies. The sensitivity of the three methods tested varied considerably, with the automatic capillary electrophoretic system being most sensitive. The latter also provides the advantage that PCRs can be scored against defined fluorescence threshold limits (this is also possible using fluorescently labelled primers and a sequencer: e.g. Harper *et al.* 2005). In gel electrophoresis, however, it can be hard to standardize whether a band is counted as ‘positive’. These advantages come at the cost of a higher price for such a system compared with standard electrophoresis. Based on the current results, we recommend agarose gel electrophoresis including gels stained with GelRed™ as a cheaper but still highly sensitive method for the visualization of PCR products. Not only is it much more sensitive than ethidium bromide, reaching nearly the standard set by QIAxcel, but this approach has the added advantage that, according to the manufacturer, it is nontoxic, which increases working safety and reduces toxic waste produced. Reducing the amount of GelRed™ to one-third of the concentration recommended by the manufacturer causes no loss in sensitivity (data not shown) and diminishes costs further.

It is known that when a PCR assay comes close to its detection limit, reproducibility of PCR results can suffer (Piggott 2004; McMillan *et al.* 2007; Seeber *et al.* 2010). This happens, for example, when only minute amounts of the template molecule are available. Within our experiment, predators that digested their meals for extended times postfeeding were repeatedly tested for prey DNA. Theses samples can be expected to contain comparably low concentrations of template prey DNA providing the opportunity to examine the reproducibility of prey detection close to the detection limit of the current PCR assays. Although we found that individual samples varied in detection success and that repeated testing resulted in an increasing total number of samples scoring positive, there was no significant variation in the average number of positives between PCRs. It is likely that if we would have used individuals killed after short digestion times and tested them for short prey DNA fragments, even less variation between PCR outcomes would have occurred for specific samples because of a sufficient number of available templates. Therefore, if (many) samples are expected to have little or highly degraded DNA, those samples yielding no PCR product should be re-screened to keep the number of false negatives to a minimum. In case large numbers of consumers are screened, retesting might not be mandatory as this variation is unlikely to affect the overall conclusions drawn from the screening results. Retesting a subsample of a set of samples can provide a good estimate on the fraction of samples containing critically low amounts of amplifiable target DNA. Assays that allow DNA detection of recently consumed food only can be advantageous for answering specific questions in trophic ecology (McMillan *et al.* 2007). However, such assays might be prone to considerable methodological variability as the percentage of consumers containing target DNA concentrations close to the detection limit might be high.

Our feeding experiments on carabid beetles and lycosid spiders show significantly higher prey detection rates in liquid feeding spiders compared with chewing predators, confirming earlier work on postfeeding prey DNA detection intervals in arthropods (Ma *et al.* 2005; Greenstone *et al.* 2007; Hosseini *et al.* 2008; Traugott & Symondson 2008). What, however, clearly distinguishes the present findings from the previous work are the very high detection rates even after long digestion times. The short- and medium-sized prey DNA fragments were detectable in all beetles up to 48 h and were missed in only three spiders up to 72-h postfeeding. Solely, the long fragments (611 and 555 bp), which are well beyond the suggested fragment length of 300 bp for prey DNA detection (King *et al.* 2008), showed a decline over time and reached 50% detection success after 30.0 and 79.2 h for beetles and spiders, respectively. This is approximately 50% longer than ∼250- to 300-bp fragments could be detected in carabid beetles fed with aphids (Sheppard *et al.* 2005) and in lycosid spiders fed *Plutella xylostella* (Hosseini *et al.* 2008). Similarly, in collembolan-fed lycosid spiders that had digested their meals for 24 h, no prey DNA amplification was possible (Kuusk & Ekbom 2010). The longer time span during which prey DNA was detectable in our experiment might be explained by a true physiological difference as we used high alpine species, which are adapted to cool environments. Still, the observed differences could as well be caused by a higher amount of ingested DNA in the present study (much less prey was offered in some of the studies mentioned earlier) or a higher sensitivity in our assays.

The present results clearly demonstrate that methodological issues affect the sensitivity and replicability of molecular gut content analysis. Whether increased assay sensitivity (i.e. sensitivity of PCR and post-PCR visualization methods) is advantageous, however, depends on the specific research questions to be answered. For example, in studies where the focal prey represents a major dietary component, maximizing assay sensitivity might not be mandatory for obtaining meaningful results. On the other hand, if predators are investigated that have a low or unknown feeding frequency, highly sensitive assays are desirable. In terms of prey detection replicability, we also propose to use highly sensitive systems to reduce the proportion of samples close to the detection limit, which is expected to increase the robustness of the screening data. Currently, assays used for the diagnosis of trophic interactions are usually not standardized for their sensitivity between studies, prohibiting the direct comparisons of trophic data reported in different papers. Future work employing molecular prey detection should thus consider and minimize the methodologically induced variation, allowing for better cross-study comparisons.

## Data accessibility

DNA sequences submitted to GenBank: accession numbers: *Acheta domesticus* (JF419327), *Tenebrio molitor* (HQ891143–HQ891145). Other data deposited at Dryad: doi: 10.5061/dryad.8947.
